# The subtleties of cognitive decline in multiple sclerosis: an exploratory study using hierarchichal cluster analysis of CANTAB results

**DOI:** 10.1186/s12883-018-1141-1

**Published:** 2018-09-10

**Authors:** Hideraldo Luis Souza Cabeça, Luciano Chaves Rocha, Amanda Ferreira Sabbá, Alessandra Mendonça Tomás, Natali Valim Oliver Bento-Torres, Daniel Clive Anthony, Cristovam Wanderley Picanço Diniz

**Affiliations:** 1 Departamento de Neurologia, Hospital Ophir Loyola, Belém, PA Brazil; 2 Laboratório de Investigações em Neurodegeneração e Infecção, Hospital Universitário João de Barros Barreto, Universidade Federal do Pará, Instituto de Ciências Biológicas, Belém, PA Brazil; 3 Faculdade de Fisioterapia e Terapia Ocupacional, Instituto de Ciências da Saúde, Universidade Federal do Pará, Belém, PA Brazil; 4 Laboratory of Experimental Neuropathology, Department of Pharmacology, University of Oxford, Oxford, UK

**Keywords:** Multiple sclerosis, Cognitive dysfunction, Reaction time, Rapid visual processing, Information processing speed, Working memory

## Abstract

**Background:**

It is essential to investigate cognitive deficits in multiple sclerosis (MS) to develop evidence-based cognitive rehabilitation strategies. Here we refined cognitive decline assessment using the automated tests of the Cambridge Neuropsychological Test Automated Battery (CANTAB) and hierarchical cluster analysis.

**Methods:**

We searched for groups of distinct cognitive profiles in 35 relapsing-remitting MS outpatients and 32 healthy controls. All individuals participated in an automated assessment (CANTAB) and in a pencil and paper general neuropsychological evaluation.

**Results:**

Hierarchical cluster analysis of the CANTAB results revealed two distinct groups of patients based mainly on the Simple Reaction Time (RTI) and on the Mean Latency of Rapid Visual Processing (RVP). The general neuropsychological assessment did not show any statistically significant differences between the cluster groups. Compared to the healthy control group, all MS outpatients had lower scores for RTI, RVP, paired associate learning, and delayed matching to sample. We also analyzed the associations between CANTAB results and age, education, sex, pharmacological treatment, physical activity, employment status, and the Expanded Disability Status Scale (EDSS). Although limited by the small number of observations, our findings suggest a weak correlation between performance on the CANTAB and age, education, and EDSS scores.

**Conclusions:**

We suggest that the use of selected large-scale automated visuospatial tests from the CANTAB in combination with multivariate statistical analyses may reveal subtle and earlier changes in information processing speed and cognition. This may expand our ability to define the limits between normal and impaired cognition in patients with Multiple Sclerosis.

**Electronic supplementary material:**

The online version of this article (10.1186/s12883-018-1141-1) contains supplementary material, which is available to authorized users.

## Background

Multiple sclerosis (MS) is a chronic inflammatory disease of the central nervous system that is associated with motor, cognitive, and neuropsychiatric symptoms that appear independently as the disease progresses [1]. Despite the high prevalence rates of cognitive dysfunction in MS, for many decades physicians and patients focused on the overt motor dysfunctions that affect the activities of daily life. It was not until 1991 that cognitive dysfunction began to be assessed in terms of its frequency, patterns, and prediction [2]. Until this time, cognitive function was not routinely assessed in patients [3–5], and the implications of cognitive deficits on the quality of life of MS patients remained unknown [6]. The prevalence of cognitive decline showed that information processing, episodic memory, and, to a lesser extent, attention and executive functions, were about 43% to 70% lower than age, sex and years of schooling matched controls [7], suggesting that several brain regions are impaired in MS. Neuroimaging continues to confirm this, and is helping define the extent and localization of areas in the central nervous system that are impaired in MS. [8, 9]

It is essential to determine the limits between normal and subtle cognitive decline in order to develop and implement clinical interventions that target cognitive rehabilitation [4] in chronic neurodegenerative diseases, including MS. In a previous report, we compared the use of the Cambridge Neuropsychological Test Automated Battery (CANTAB) and language tests to detect subtle differences in cognitive performance in two age groups. To distinguish the limits between normal and abnormal cognitive decline as age progresses we suggested, as an alternative to language tests, large-scale application of automated visuospatial cognitive tests [10].

The CANTAB is a nonverbal visuospatial stimulus battery that uses touchscreen technology to obtain nonverbal responses from participants. This is in line with recent recommendations to use more precise automated neuropsychological tests in MS. [11] Both longitudinal and cross-sectional studies have shown that the CANTAB is particularly well suited for cognitive assessments of patients from various cultures as it involves minimal interference from the researcher or clinician during data acquisition [12].

In this study, we aimed to utilize the CANTAB with multivariate analysis to assess cognitive function in MS patients to investigate the performance limits in cognitively impaired and unimpaired subjects as compared to control groups. A few studies have used the CANTAB to measure cognitive decline in MS patients [13–18], but none have searched for subgroups of patients with different patterns of cognitive impairment using multivariate statistical procedures. We hypothesized that there may be distinct subgroups of MS patients based on cognitive decline and that hierarchical cluster analysis of CANTAB results may be able to detect such groups. We expect that an improved understanding of cognitive deficits in MS could help guide evidence-based cognitive rehabilitation programs, and the selection of therapy, based on the cognitive profiles of MS patients [19, 20].

## Methods

This observational exploratory study investigated whether the CANTAB in combination with hierarchical cluster analysis could detect subtle cognitive declines in MS to classify MS patients according to their performance on selected CANTAB tests. All subjects provided informed written consent prior to their participation, in accordance with the Declaration of Helsinki, which was voluntary. Patient data were coded to preserve confidentiality. This study was approved by the local ethics committee (Comitê de Ética em Pesquisa do Hospital Universitário João de Barros Barreto, protocol number 2.160.639), and it followed the International Ethical Guidelines for Health-related Research involving Humans (CIOMS/WHO).

### Subjects

Thirty-five outpatients diagnosed with relapsing-remitting MS subtype (revised McDonald criteria, 2010) [21] were invited to participate. MS patients from a demyelinating clinic of a tertiary hospital were invited to participate. The inclusion criteria limited the studied group to MS relapsing-remitting subtype patients (revised McDonald criteria, 2010), less than 60 years old age, visual acuity (20/20 in Snellen’s test) and at least eight years of formal education. Patients with previous cranioencephalic trauma, stroke, dementia, or other neurological diseases including past or actual criteria for primary depression (DSM IV) were excluded.

### Study design

All of the MS participants, who were in remission at the time of testing, and all of the control subjects met the inclusion criteria, participated in a standardized pencil and paper neuropsychological assessment as well as the CANTAB on a single day. The neuropsychological assessment results were subjected to an initial cluster analysis limited to multimodal variables, resulting in the formation of a selected multiple sclerosis group (MS group, with only MS patients), healthy control group (HC group, with only healthy control subjects) and Group 1 and Group 2 (where MS and HC appeared together in the same cluster). To investigate the influence of exercise and employee as significant variables that may change cognitive assessment results we defined as exercised individuals, those practicing exercise for at least six months, three times a week, and as employed subjects, those citizens that work in any job for, at least, six months.

### Standard neuropsychological assessment

The standard pencil and paper neuropsychological assessment was adjusted for use in a Brazilian population, including the Mini-Mental State Examination, the Verbal Fluency test and the Word List Memory, Recall, and Recognition tests [22]. Trained investigators administered these tests in about 30–45 min in an environment that had adequate lighting and reduced noise conditions.

### Automated neuropsychological assessment (the CANTAB)

The three cognitive domains explored by the CANTAB are working memory and planning; attention; and visuospatial memory. All the tests in the battery utilize touchscreen responses, which minimizes potential interference through verbal instruction. All participants were assessed individually. The assessment started with a motor screening task to introduce the CANTAB touchscreen basic procedure. This task gives a general idea of potential sensorimotor or other difficulties that could limit valid data collection. After they become familiar with the touchscreen procedure, each participant was assessed on the following tasks: Rapid Visual Information Processing (RVP), which measures sustained attention; Reaction Time (RTI), which reflects motor and mental response speeds as well as movement time, reaction time, response accuracy, and impulsivity; Paired Associate Learning (PAL), which assesses visual memory and new learning; Spatial Working Memory (SWM), which measures the retention and manipulation of visuospatial information; and Delayed Matching to Sample (DMS), which, through forced choice, assesses recognition memory of visual patterns and tests both simultaneous matching and short-term visual memory. All battery generally lasts between 30 and 60 min, depending on the subject’s performance. Additional file 1: Table S1 describes the cognitive tests based on the CANTAB user manual. For further details of the neuropsychological test, please see: http://www.cambridgecognition.com/cantab/cognitive-tests/.

### Data analysis

We analyzed all data using Biostat 5.3®, Statistica 7®, and Graphpad Prism® software. Continuous variables are represented as means and standard deviations, and *p* values lower than 0.05 were considered significant. The statistical tests for intergroup comparisons included Student’s t test for normally distributed data or the Mann-Whitney test for non-parametric analysis. A correlation matrix was used to assess potential associations between variables inside or between groups. All quantitative variables were submitted to an initial cluster analysis (Ward’s method, Euclidean distance). We applied this multivariate statistical procedure to our sample of behavioral data to search for possible group of patients sharing similar performances. The classes suggested by cluster analysis were assessed by a forward stepwise discriminant function analysis. Discriminant function analysis classifies and predicts the probability of unknown individuals to be classified into a certain group indicating the variables that best contributed to group formation. It assumes that the sample is normally distributed and as such, uses these variables to determine whether groups differ about the mean of a variable. The purpose of the analysis is to learn how one can discriminate between potential groups of distinct cognitive performances, based on the scores of each individual test results. Hierarchical cluster analysis (Ward’s method and Euclidian distances) used multimodal or at least bimodal distributions. We measured the relative contribution of each variable for cluster formation using discriminant analysis.

We also expressed the results as Z-scores which is the number of standard deviations from the mean a data point is, which allows to compare the results with a normal distribution.

## Results

### Multiple sclerosis patients profile

The mean age of MS patients was 34.2 ± 10 years (range: 18–55) with mean education years of 13.8 ± 3.5 years (range: 8–23), mean Expanded Disability Status Scale (EDSS) score of 1.44 ± 1.4 (median: 1; range: 0–6), average duration of disease of 4.66 ± 4 years (range: 0.25–13.6) and average acute exacerbations of 1.82 ± 0.5 times (range: 1–3). Thus, this MS group consists of mostly patients in the early years of their disease and disability.

In this study cohort, which comprised an MS group (*n* = 35) and a healthy control (HC) group (*n* = 32), most of the participants were female. In the MS group, subcutaneous (44 μg) or intramuscular interferon β-1a was the main disease-modifying drug therapy. Others included subcutaneous interferon β-1b, subcutaneous glatiramer acetate (20 mg), intravenous Natalizumab and none. There were no significant intergroup differences in age and education (*p* > 0.05, Student’s t test).

Table 1 shows the descriptive demographic data as absolute values and percentages, and Table 2 shows the descriptive performance data as means and standard deviations and effect sizes (Cohen’s d, Hedges’ g and Glass’ delta for variables with high variance).

**Table 1:** Descriptive demographic data for the Multiple Sclerosis (MS) and Healthy Control groups

	Multiple Sclerosis	Healthy Control
N	35	32
Age (years)	34.2 ± 10 (18–55)	32.03 ± 8.40
Education (years)	13.8 ± 3.5 (8–23)	14.70 ± 3.42
Expanded Disability Status Scale (EDSS) score	1.44 ± 1.4 (0–6)	–
Average duration of disease (years)	4.66 ± 4 (0.25–13.6)	–
Average acute exacerbations (n)	1.82 ± 0.5 (1–3)	–
SEX		
Men (n)	6 (17%)	9 (28%)
Women (n)	29 (83%)	23 (72%)
Pharmacologic treatment		
Interferon-β 1a (n)	16 (45.7%)	–
Interferon-β 1b (n)	8 (22.85%)	–
Glatiramer acetate (n)	3 (8.6%)	–
Natalizumabe (n)	2 (5.7%)	–
None (n)	6 (17.15%)	32 (100%)
Physical activity		
Exercised (n)	9 (25.7%)	12 (37.5%)
Sedentary (n)	26 (74.3%)	20 (62.5%)
Employment status		
Yes (n)	28 (80%)	32 (100%)
No (n)	7 (20%)	0 (0%)

**Table 2:** Performances of Multiple Sclerosis (MS) and Healthy Control groups and intergroup effects’ sizes (Cohen’s d, Hedge’s G and Glass’ Δ for high variances values). Values are shown as mean and standard deviation. Effects’ sizes with significant T Student’s Test or Mann-Whitney Test (*p* < 0.05) are identified with *

	Multiple Sclerosis	Healthy Control	Cohen's D	Hedge's G	Glass' Δ
Spatial Working Memory (SWM)					
Strategy (STG)	38.28 ± 3.63	35.71 ± 6.95	0.487*	0.494*	0.388*
Total Errors (TE)	49.11 ± 20.22	41.56 ± 23.55	0.325	0.327	0.296
Rapid Visual Processing (RVP)					
A’	0.84 ± 0.06	0.88 ± 0.04	0.766*	0.759*	1.012*
Probability of Hit (PH)	0.49 ± 0.16	0.57 ± 0.16	0.401	0.409	0.433
Mean Latency (ML)	567.67 ± 167.17	446.19 ± 72.97	1.115*	1.099*	1.790*
Paired Associate Learning (PAL)					
First Trial Memory Score (FTMS)	11.8 ± 4.43	13.18 ± 3.02	4.623	0.396	0.517
Mean Trials to Success (MTS)	3.17 ± 1.68	2.07 ± 0.76	0.870*	0.857*	1.486*
Total Errors Adjusted (TEA)	37.71 ± 35.62	17.84 ± 14.28	0.776*	0.763*	1.597*
Reaction Time (RTI)					
5-Choice Accuracy Score (5CAS)	14.71 ± 0.62	14.93 ± 0.24	0.483	0.475	0.932
5-Choice Movement Time (5CMT)	675.92 ± 182.6 ms	598.55 ± 131.31 ms	0.655	0.649	0.862
5-Choice Reaction Time (5CRT)	446.31 ± 94.19 ms	403.93 ± 76.61 ms	0.424	0.422	0.474
Simple Accuracy Score (SAS)	14.54 ± 0.95	14.81 ± 0.47	0.223*	0.223*	0.221*
Simple Movement Time (SMT)	688.01 ± 198.74 ms	663.33 ± 220.78 ms	0.267	0.267	0.258
Simple Reaction Time (SRT)	425.19 ± 87.09 ms	377.23 ± 76.04 ms	0.657*	0.654*	0.699*
Delayed Matched to Sample (DMS)					
Total Correct (TC)	16.31 ± 2.98	17.96 ± 1.46	0.660*	0.650*	1.141*
Mini-Mental State Examination (MMSE)	28.17 ± 2.35	29.25 ± 0.85	0.611	0.600	1.270
Verbal fluency 1 (ANIMALS)	15.65 ± 5.72	18.61 ± 3.68	0.615*	0.610*	0.804*
Verbal fluency 2 (FRUITS)	14.54 ± 4.06	16.74 ± 3.24	0.599*	0.596*	0.679*
Verbal fluency 3 (A)	10.94 ± 4.89	12.96 ± 4.33	0.437*	0.436*	0.466*
Verbal fluency 4 (F)	12.37 ± 4.9	16.09 ± 3.76	0.852*	0.847*	0.989*
Word list Memory task	20.2 ± 3.27	20.51 ± 3.43	0.092	0.093	0.090
Word list recall	6.85 ± 1.73	7.16 ± 1.63	0.184	0.184	0.190
Word list recognition	9.12 ± 1.07	9.51 ± 0.99	0.378	0.378	0.394

### Cognitive performance in the MS and HC groups

**Fig. 1:**
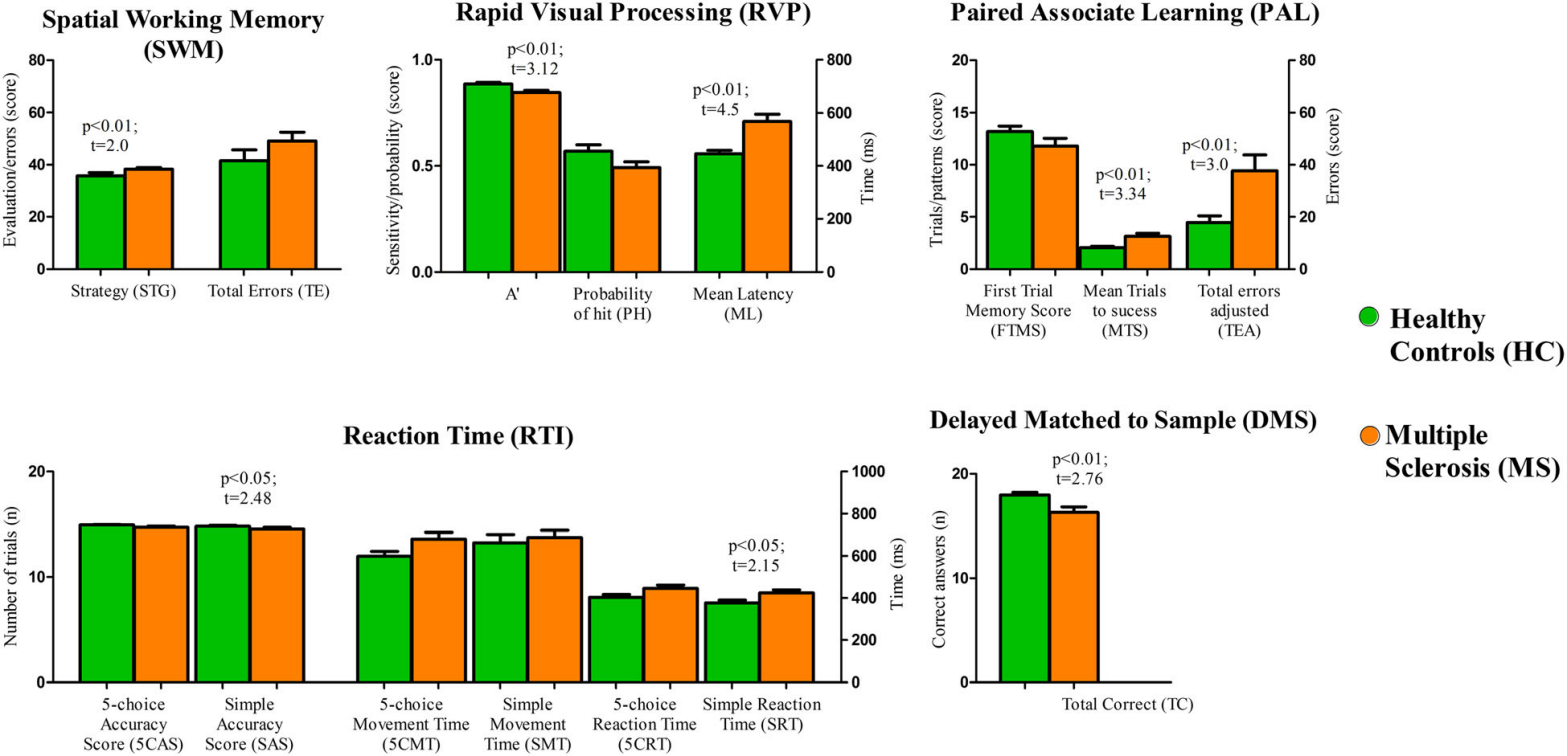
CANTAB performance scores in the Multiple Sclerosis and Healthy Control groups. The *p*-values were obtained using Student’s t test or the Mann-Whitney test. The Spatial Working Memory (SWM) test measures the retention and manipulation of visuospatial information; the Rapid Visual Processing (RVP) test measures sustained attention; the Paired Associate Learning (PAL) test assesses visual memory and new learning; the Reaction Time (RTI) test reflects motor and mental response speeds as well as movement time, reaction time, response accuracy, and impulsivity; and the Delayed Matched to Sample (DMS) test uses forced choice to assess the recognition memory of visual patterns and tests both simultaneous matching and short-term visual memory

The MS and HC groups had significantly different mean scores on CANTAB tests, with the Spatial Working Memory (SWM) being the exception. Table 2 and Fig. 1 show that the MS group had lower average scores than the HC group. Table 2 also shows the Effects’ sizes (Cohen’s d, Hegdes’ g and Glass’ Δ) of intergroup disparities by Student’s t test and Mann-Whitney test to quantify these performance differences. These findings reflect the impairment of a variety of cognitive domains.

### Age, education, pharmacological treatment, physical activity, sex, and employment status all influenced the results of intergroup analysis

Table 3 shows descriptive data (score means and correspondent standard deviations) of all neuropsychological tests scores of Groups 1 and 2. There were, respectively, significant positive and negative correlations between age, education, and EDSS and the CANTAB tests, with r values ranging from -0.478 to 0.532. Age and education correlated significantly with Reaction Time (RTI), Paired Associate Learning (PAL), and Delayed Matched to Sample tests. Yet, education alone correlated with all CANTAB tests, including Rapid Visual Processing (RVP) and EDSS score correlated with SWM and DMS tests scores. Table 4 summarizes all the correlations results reported with *p*-values and correlation coefficients.

**Table 3:** Test performance descriptive data, represented as means and standard deviations for Group 1 and Group 2. Values are shown as mean and standard deviation

	GROUP 1 (MEAN ± SD, *N* = 44)	GROUP 2 (MEAN ± SD, *N* = 23)
Spatial Working Memory (SWM)		
Strategy (STG)	36.06 ± 5.9	38.95 ± 4.43
Total Errors (TE)	38.9 ± 21.37	58.13 ± 17.61
Rapid Visual Procesing (RVP)		
A’	0.88 ± 0.04	0.82 ± 0.06
Probability of Hit (PH)	0.56 ± 0.16	0.46 ± 0.15
Mean Latency (ML)	442.03 ± 71.71	639 ± 158.80
Paired Associate Learning (PAL)		
First Trial Memory Score (FTMS)	13.77 ± 3,14	9.95 ± 3.91
Mean Trials to Success (MTS)	2.06 ± 0.82	3.77 ± 1.68
Total Errors (TEA)	17.47 ± 15.08	48.78 ± 37.83
Reaction Time (RTI)		
5-choice Accuracy Score (5CAS)	584.12 ± 115.41	746.96 ± 197.67
5-choice Movement Time (5CMT)	584.13 ± 115.42 ms	750.6 ± 194 ms
5-choice Reaction Time (5CRT)	392.37 ± 54.5 ms	490.54 ± 104.51 ms
Simple Accuracy Score (SAS)	14.8 ± 0.51	14.43 ± 1.08
Simple Movement Time (SMT)	599.33 ± 161.57 ms	823.33 ± 211.45 ms
Simple Reaction Time (SRT)	359.91 ± 43.02 ms	483.32 ± 86.85 ms
Delayed Matched to Sample (DMS)		
Total Correct (TC)	18.25 ± 1.33	14.91 ± 2.79
Mini-Mental State Examination (MMSE)	28.04 ± 4.01	27.65 ± 2.7
Verbal fluency 1 (ANIMALS)	18.81 ± 4.15	13.73 ± 5.02
Verbal fluency 2 (FRUITS)	16.67 ± 3.46	13.52 ± 3.71
Verbal fluency 3 (A)	13.04 ± 4.59	9.74 ± 4.23
Verbal fluency 4 (F)	15.67 ± 4.03	11.21 ± 4.7
Word list memory task	20.65 ± 3.75	19.78 ± 2.31
Word list recall	7.07 ± 1.65	6.87 ± 1.76
Word list recognition	9.39 ± 0.95	9.15 ± 1.21

**Table 4:** Correlation Matrix (Spearman Rank Order Correlations) with Age, Education, EDSS score and CANTAB tests’ measures. Correlations in bold are statistically significant (*p* < 0.05) with *r* values shown ranging from −0.884 to 0.959. Note that Age, Education and EDSS score had only mild to moderate correlations with CANTAB tests’ measures (*r* values ranging from -0.478 to 0.532). Abbreviations utilized from the List of Abbreviations in this paper

	**AGE**	**ED**	**EDSS**	**STG**	**TE**	**A’**	**PH**	**ML**	**FTMS**	**MTS**	**TEA**	**FCAS**	**5CMT**	**5CRT**	**SAS**	**SMT**	**SRT**	**TC**
**AGE**	1.000	− 0.034	0.271	**0.333**	**0.429**	−0.137	− 0.074	− 0.144	**− 0.478**	**0.502**	**0.502**	− 0.079	0.207	0.066	− 0.084	**0.383**	0.169	**−0.389**
**ED**	−0.034	1.000	−0.166	−0.189	**− 0.392**	**0.532**	**0.456**	0.225	**0.304**	**−0.267**	**−0.267**	**0.276**	−0.197	− 0.192	0.194	**− 0.297**	**−0.353**	**0.367**
**EDSS**	0.271	−0.166	1.000	**0.428**	**0.384**	−0.189	−0.254	− 0.254	−0.191	0.301	0.241	0.133	0.302	0.080	−0.123	0.151	0.166	**−0.396**
**STG**	**0.333**	−0.189	**0.428**	1.000	**0.750**	−0.191	−0.098	− 0.192	**−0.280**	**0.266**	**0.307**	−0.014	**0.321**	0.105	−0.157	**0.447**	**0.263**	**−0.311**
**TE**	**0.429**	**−0.392**	**0.384**	**0.750**	1.000	**−0.278**	−0.135	− 0.238	**−0.422**	**0.392**	**0.401**	−0.219	0.237	0.097	−0.184	**0.401**	**0.260**	**−0.308**
**A’**	−0.137	**0.532**	−0.189	−0.191	**− 0.278**	1.000	**0.891**	**0.407**	**0.393**	**−0.427**	**−0.439**	**0.324**	−0.168	− 0.135	**0.397**	**− 0.302**	**−0.299**	**0.550**
**PH**	−0.074	**0.456**	−0.254	−0.098	− 0.135	**0.891**	1.000	**0.385**	**0.243**	**−0.259**	**−0.275**	**0.276**	−0.111	− 0.126	**0.281**	− 0.212	−0.233	**0.406**
**ML**	−0.144	0.225	−0.254	−0.192	− 0.238	**0.407**	**0.385**	1.000	0.165	**−0.281**	**−0.246**	**0.292**	−0.200	− **0.259**	**0.251**	− 0.093	**−0.380**	**0.314**
**FTMS**	**−0.478**	**0.304**	−0.191	**−0.280**	**− 0.422**	**0.393**	**0.243**	0.165	1.000	**−0.850**	**−0.884**	**0.305**	−0.205	− 0.005	0.187	**− 0.378**	−0.182	**0.489**
**MTS**	**0.502**	**−0.267**	0.301	**0.266**	**0.392**	**−0.427**	**−0.259**	**− 0.281**	**−0.850**	1.000	**0.959**	**−0.242**	0.216	−0.005	**−0.261**	**0.348**	0.152	**−0.546**
**TEA**	**0.502**	**−0.267**	0.241	**0.307**	**0.401**	**−0.439**	**−0.275**	**− 0.246**	**−0.884**	**0.959**	1.000	**−0.241**	**0.276**	−0.020	**−0.243**	**0.407**	0.136	**−0.540**
**FCAS**	−0.079	**0.276**	0.133	−0.014	−0.219	**0.324**	**0.276**	**0.292**	**0.305**	**−0.242**	**−0.241**	1.000	−0.149	**− 0.308**	**0.307**	− 0.124	**−0.289**	0.222
**5CMT**	0.207	−0.197	0.302	**0.321**	0.237	−0.168	−0.111	− 0.200	−0.205	0.216	**0.276**	−0.149	1.000	**0.270**	−0.221	**0.776**	**0.294**	**−0.303**
**5CRT**	0.066	−0.192	0.080	0.105	0.097	−0.135	−0.126	**− 0.259**	−0.005	− 0.005	−0.020	**− 0.308**	**0.270**	1.000	**−0.257**	0.235	**0.795**	**−0.414**
**SAS**	−0.084	0.194	−0.123	−0.157	− 0.184	**0.397**	**0.281**	**0.251**	0.187	**−0.261**	**−0.243**	**0.307**	−0.221	**− 0.257**	1.000	− 0.098	−0.116	**0.297**
**SMT**	**0.383**	**−0.297**	0.151	**0.447**	**0.401**	**−0.302**	−0.212	− 0.093	**−0.378**	**0.348**	**0.407**	−0.124	**0.776**	0.235	−0.098	1.000	**0.421**	**−0.400**
**SRT**	0.169	**−0.353**	0.166	**0.263**	**0.260**	**−0.299**	−0.233	**− 0.380**	−0.182	0.152	0.136	**−0.289**	**0.294**	**0.795**	−0.116	**0.421**	1.000	**−0.506**
**TC**	**−0.389**	**0.367**	**−0.396**	**−0.311**	**− 0.308**	**0.550**	**0.406**	**0.314**	**0.489**	**−0.546**	**−0.540**	0.222	**−0.303**	**− 0.414**	**0.297**	**− 0.400**	**−0.506**	1.000

Notably, unemployed subjects had lower scores for SWM, A’ (RVP), and PH (one-way ANOVA; *p* < 0.05, *p* < 0.01, and *p* < 0.01, respectively). Subjects who were being treated with interferon β-1a and β-1b based therapy (*n* = 24) did not impact on outcome compared to subjects who were being treated with other therapy or who were not being treated with any reported medication (*n* = 11; one-way ANOVA, *p* > 0.05).

### Multivariate analysis: Multimodal index, hierarchical cluster analysis, and discriminant analysis

**Fig. 2:**
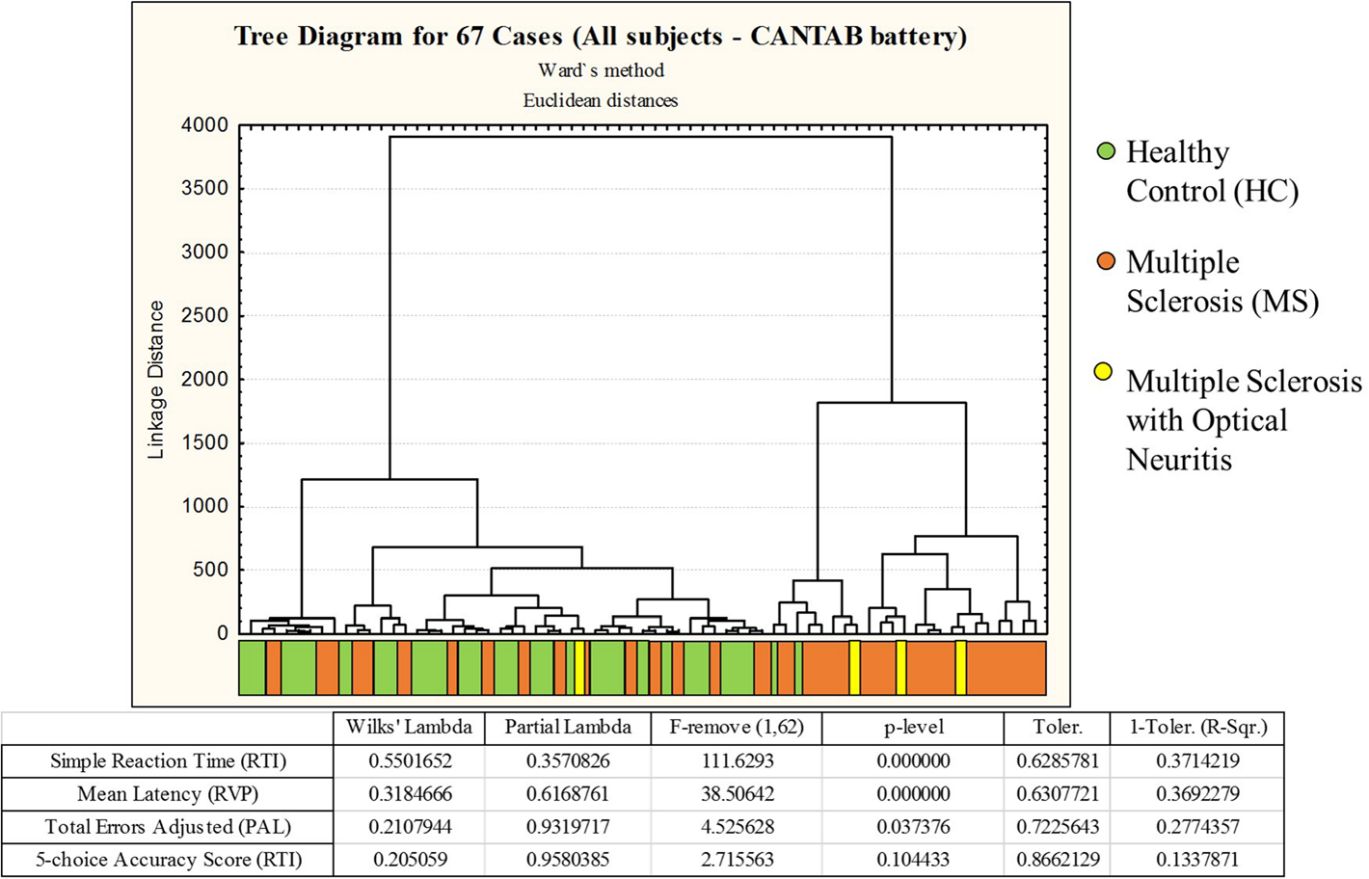
Dendrograms from a cluster analysis of healthy control subjects and multiple sclerosis patients based on their performance on selected CANTAB tests. This analysis identified two main clusters based on test performance, with multimodal variables contributing to different extents to cluster formation. Healthy control subjects and some multiple sclerosis patients are grouped on the left side (Group 1), and another group of multiple sclerosis patients and some healthy control subjects are grouped on the right (Group 2). Discriminant analysis results are shown in the table below the dendrogram, as are the probability density values (p-levels) that were used to identify which neuropsychological tests contributed most to cluster formation

**Fig. 3:**
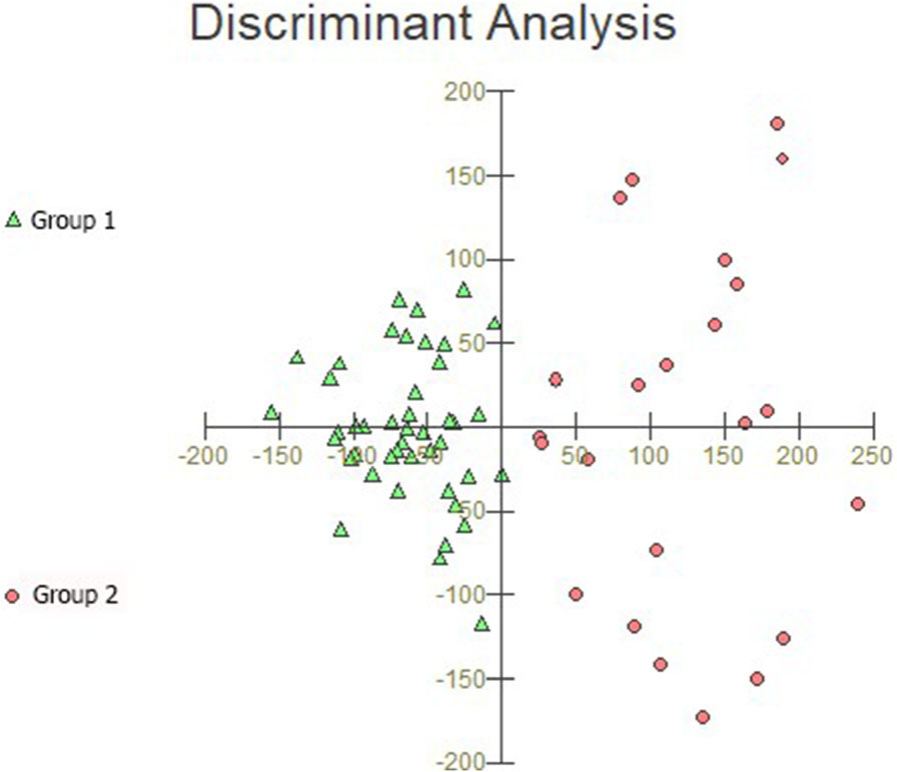
Graphic representation of discriminant analysis using the same dataset as in Fig. 2. Note the smaller dispersion of Group 1 performances, which suggests that healthy controls and a subgroup of multiple sclerosis patients had similar cognitive performance. In contrast, Group 2 performances show greater dispersion for selected CANTAB neuropsychological tests, with the spatial distribution for Group 2 being quite distinct from that of the Group 1 dataset

Cluster analyses were performed using either a combination of general neuropsychological assessment data and CANTAB test results or using CANTAB results alone. Only bimodal or multimodal variables (Multimodal index> 0.5) were selected for cluster analysis (see Schweitzer and Renehan [23] for details). Thus, the following variables were used for the general hierarchical cluster analysis: mean latency (ML), mean trials to success (MTS), total errors adjusted (TEA), 5-choice accuracy score (5CAS), 5-choice reaction time (5CRT), simple accuracy score (SAS), and simple reaction time (SRT) from the CANTAB; the Mini-Mental State Examination (MMSE); and the Word List Recognition (WLR). In addition, we performed a separate cluster analysis that was limited to CANTAB variables. The results of the cluster analyses were similar when we used the dataset of multimodal variables of the general neuropsychological assessment + CANTAB and when we used the dataset that was limited to CANTAB variables. However, almost only CANTAB variables contributed to cluster formation in the general assessment, so we decided to limit the subsequent analysis to the CANTAB dataset. This analysis distinguished two groups based on test results: Group 1, which included control subjects and a subset of MS patients, and Group 2, which comprised mostly of MS patients and a few control subjects (Figs. 2 and 3). Figure 3 exhibits X-Y plot of the discriminant analysis results related to the data set of Fig. 2.

Discriminant analysis of the dataset in Fig. 2 revealed that the RTI test was the variable that contributed most to cluster formation, showing that RTI could easily differentiate the cognitive status of MS patients. In addition, the ML of the RVP test, which is a reaction time measurement based on the median latency response after recognition of a sequence of visual stimuli, could also discriminate between Groups 1 and 2. This confirmed that the most significant change in these MS patients was a reduction in information processing speed (IPS). Although it had a more limited influence, Total Errors (adjusted) from PAL test also discriminated between Groups 1 and 2 (please see the table under the dendrogram in Fig. 2, as well as Fig. 3 for details). Pharmacological treatment, physical activity, employment status, and sex did not map to the Group 1 and Group 2 distribution patterns in.

**Fig. 4:**
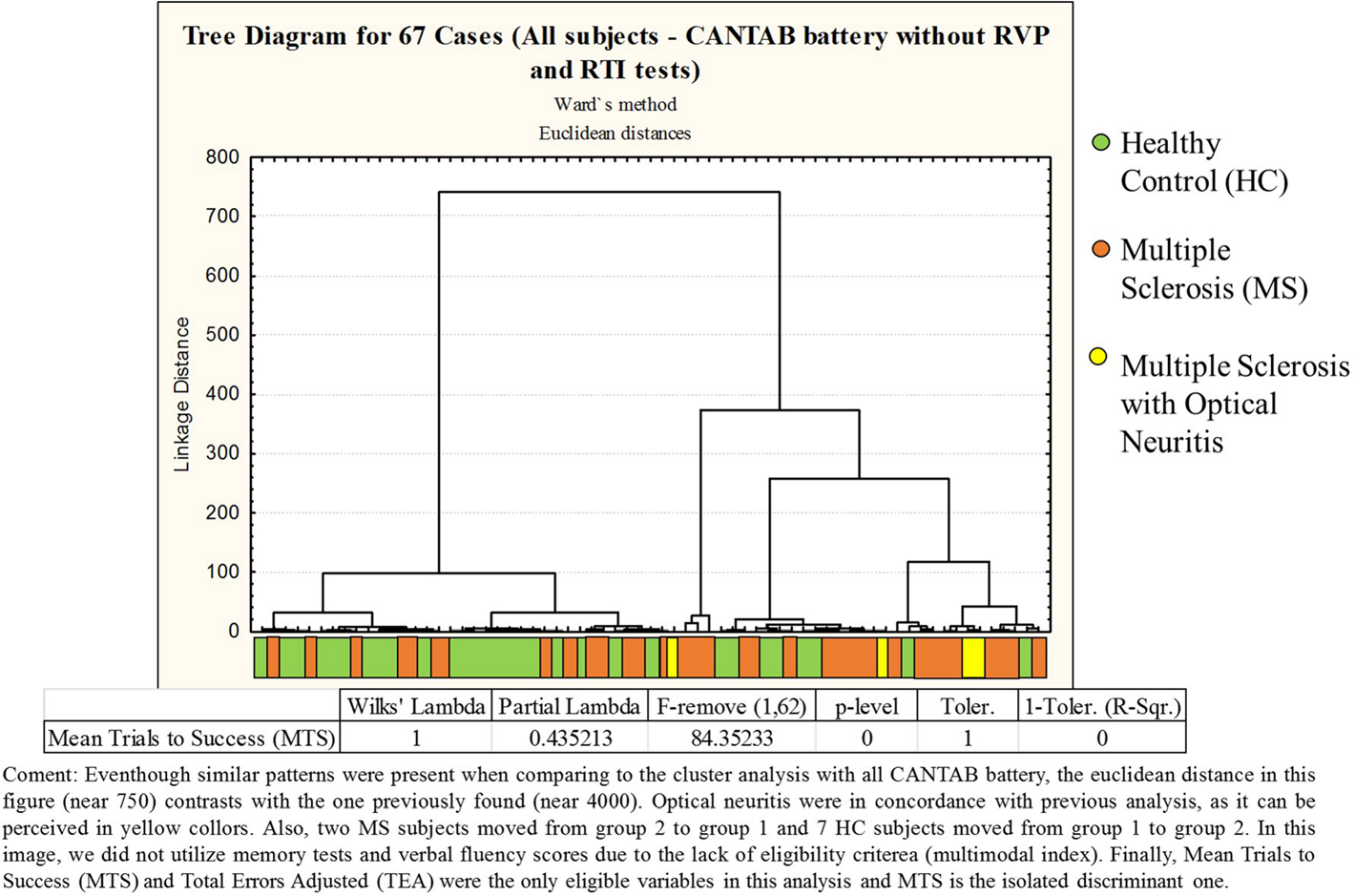
Dendrograms from a cluster analysis of healthy control subjects and multiple sclerosis patients based on their performance on selected CANTAB tests without Rapid Visual Processing (RVP) and Reaction Time (RTI) scores. Even though similar patterns were present when comparing to the cluster analysis with all CANTAB battery, the Euclidean distance in this figure (near 750) contrasts with the one previously found (near 4000). Optical neuritis were in concordance with previous analysis, as it can be perceived in yellow colors. Also, two MS subjects moved from group 2 to group 1 and 7 HC subjects moved from group 1 to group 2. In this image, we did not utilize memory tests and verbal fluency scores due to the lack of eligibility criteria (multimodal index). Finally, Mean Trials to Success (MTS) and Total Errors Adjusted (TEA) were the only eligible variables in this analysis and MTS is the isolated discriminant one

**Fig. 5:**
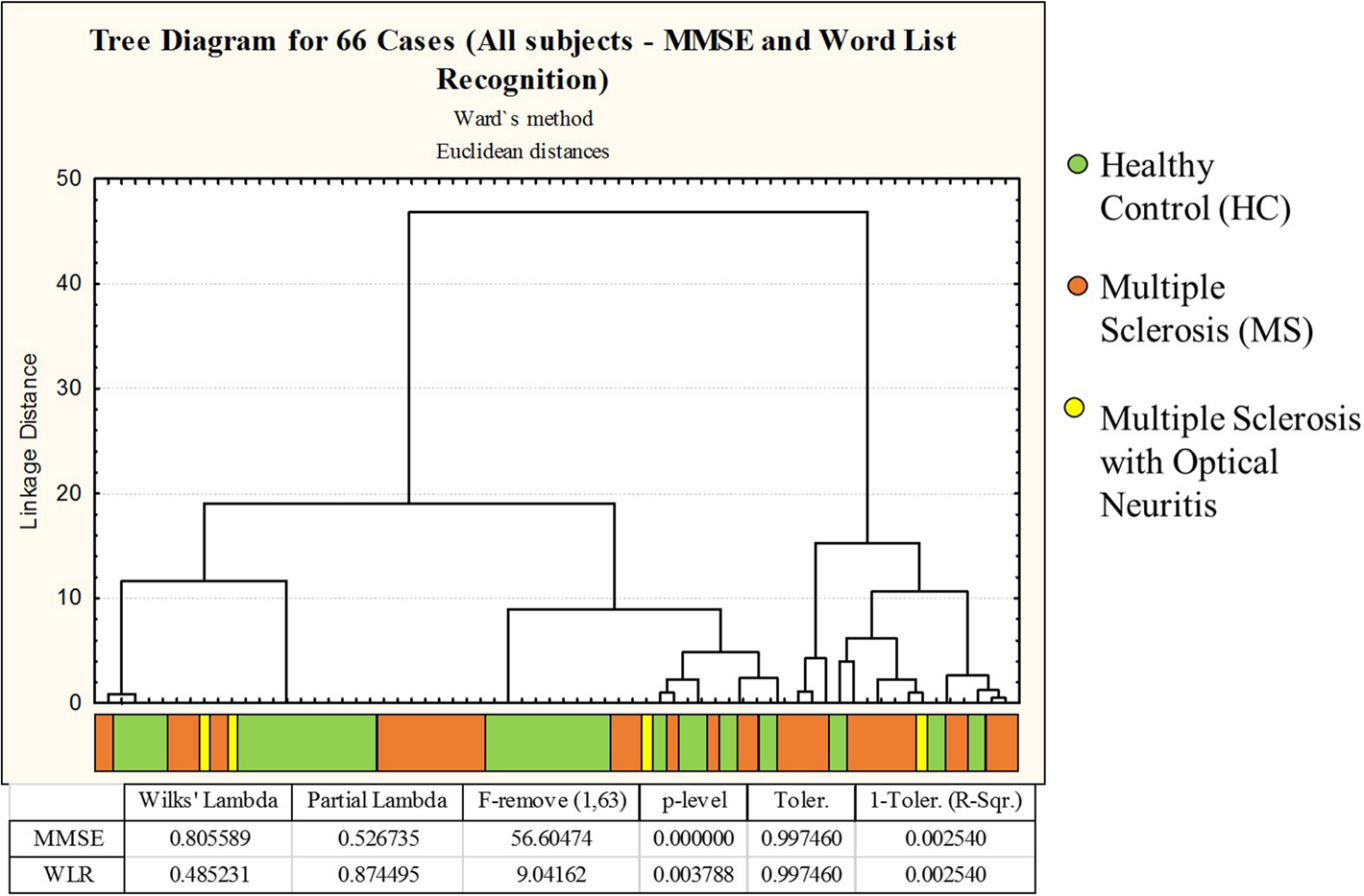
Dendrograms from a cluster analysis of healthy control subjects and multiple sclerosis patients based on their performance on Mini-Mental State Examination (MMSE) and Word List Recognition (WLR). The Euclidean distance in this figure (near 50) highly contrasts with the analysis with only CANTAB battery (near 4000). Three subjects with optical neuritis move from group 2 to group 1. Some MS patients in group 2 of the previous analysis (CANTAB) migrated to group 1 in this analysis, diminishing the size of group 2. Finally, Mini-Mental State Examination (MMSE) and Word List Recognition (WRL) were the only analyzed variables and MMSE is the one that contributes most to group differentiation

Yet, we also utilized cluster analysis without RVP and RTI tests (Fig. 4) and with only MMSE and language tests (Fig. 5), resulting in group formation with lower Euclidean distances.

**Fig. 6:**
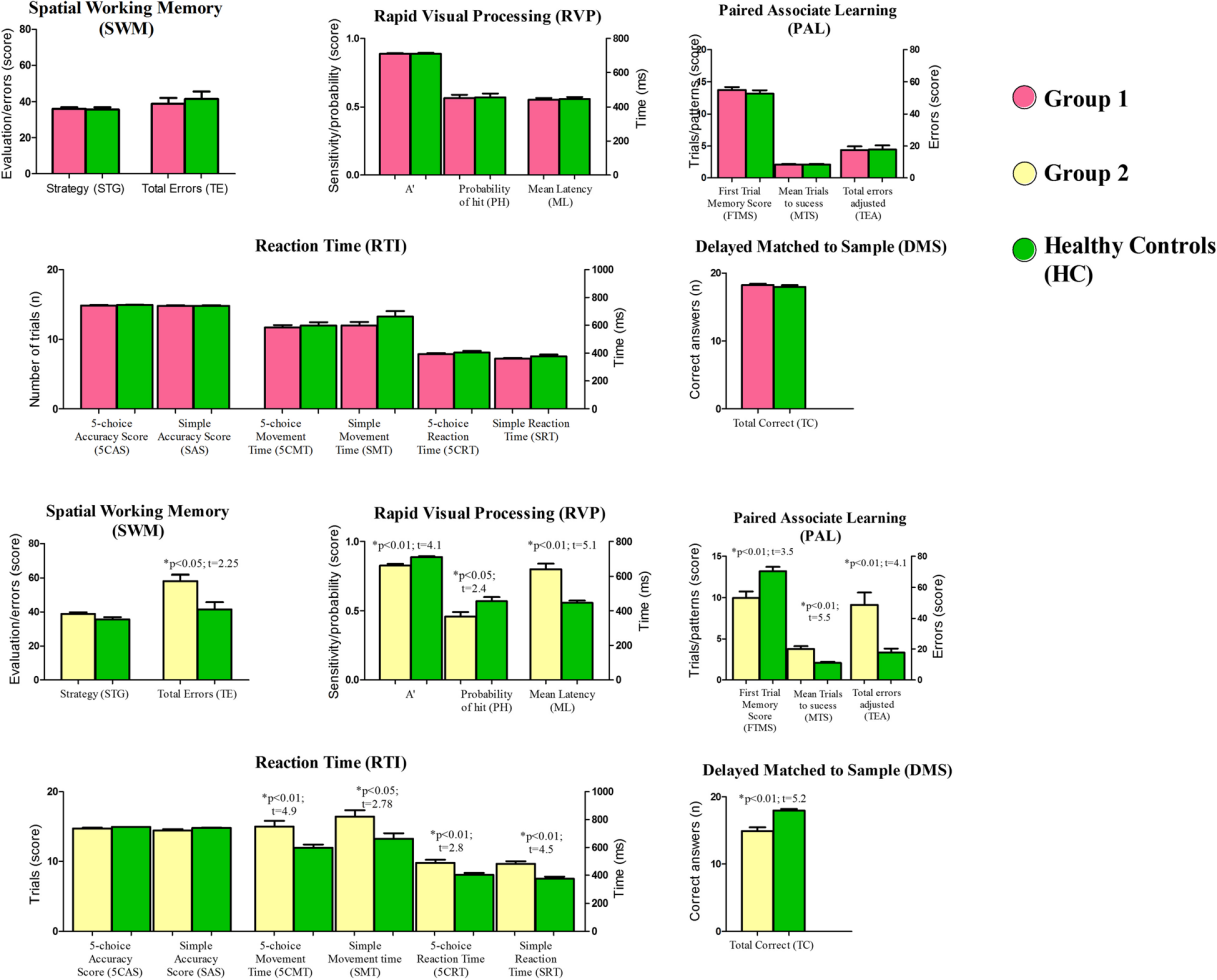
CANTAB performance scores for Group 1 and Group 2 as compared with healthy controls based on CANTAB battery cluster analysis. Statistically significant differences were set as *p* < 0.05 using Student’s t test or the Mann-Whitney test. Even though Group 1 included a number of multiple sclerosis patients, there were no significant differences between the healthy control group and Group 1, suggesting that the multiple sclerosis patients in Group 1 were not significantly different than subjects in the healthy control group. In contrast, compared to the healthy control group, Group 2 showed significantly lower performance, particularly on CANTAB tasks that relied on rapid information processing

Table 5 shows quantitative summary of z-score cognitive deficits based on means of Healthy Control (HC) group. Fig. 6 shows graphs that illustrate the differences and similarities between Group 1 and Group 2. Group 2, but not Group 1 showed significantly lower performance than the HC Group. The SWM scores of the MS Group were not significantly different than those of the HC Group; however, Group 2 showed lower scores than Group 1, which suggests that cluster analysis of the Groups that is based on CANTAB results of multimodal variables could detect subtle cognitive deficits that were previously undetectable using pencil and paper general neuropsychological assessment.

**Table 5:** Quantitative summary of Multiple Sclerosis patients z-score cognitive deficits based on means of Healthy Control (HC) group performance. All variables showed statistical intergroup difference (T test or Mann Whitney test) between Multiple Sclerosis (MS) and Healthy Control (HC) groups. Variables with significant outcomes in discriminant analysis are identified with (*). In addition, tests’ measures of Information Processing Speed (IPS) are marked with blue color and memory tests’ scores, in general, are marked with orange color. Note that almost all subjects showed, at least, subtle cognitive deficits (91.4%) in information processing speed, and most of them showed, at least, subtle cognitive deficit (71.4%) in memory. Only one subject did not present any cognitive deficit based on z-scores. Abbreviations utilized from the List of Abbreviations in this paper

Analyzed Measures	Z-score Deficits	N (%)
All Measures	≥0.5	34 (97.1%)
	≥1.0	30 (85.7%)
	≥1.5	23 (65.7%)
Simple Reaction Time (SRT, RTI test)*	≥0.5	19 (54.3%)
	≥1.0	8 (22.8%)
	≥1.5	7 (20%)
Mean Latency (ML, RVP test)*	≥0.5	22 (62.9%)
	≥1.0	20 (57.1%)
	≥1.5	14 (40%)
Total Errors Adjusted (TEA, PAL test)*	≥0.5	21 (60%)
	≥1.0	14 (40%)
	≥1.5	11 (31.4%)
A’ (RVP)	≥0.5	22 (62.8%)
	≥1.0	15 (42.8%)
	≥1.5	11 (31.4%)
Mean Trials to Success (MTS, PAL test)	≥0.5	18 (51.4%)
	≥1.0	16 (45.7%)
	≥1.5	13 (37.1%)
Simple Accuracy Score (SAS, RTI test)	≥0.5	11 (31.4%)
	≥1.0	11 (31.4%)
	≥1.5	11 (31.4%)
Total Correct (TC, DMS test)	≥0.5	19 (54.3%
	≥1.0	15 (42.9%)
	≥1.5	12 (34.3%)
Information Processing Speed (IPS)	≥0.5	32 (91.4%)
	≥1.0	28 (80%)
	≥1.5	22 (62.8%)
Memory (General)	≥0.5	25 (71.4%)
	≥1.0	20 (57.1%)
	≥1.5	16 (42.8%)

**Fig. 7:**
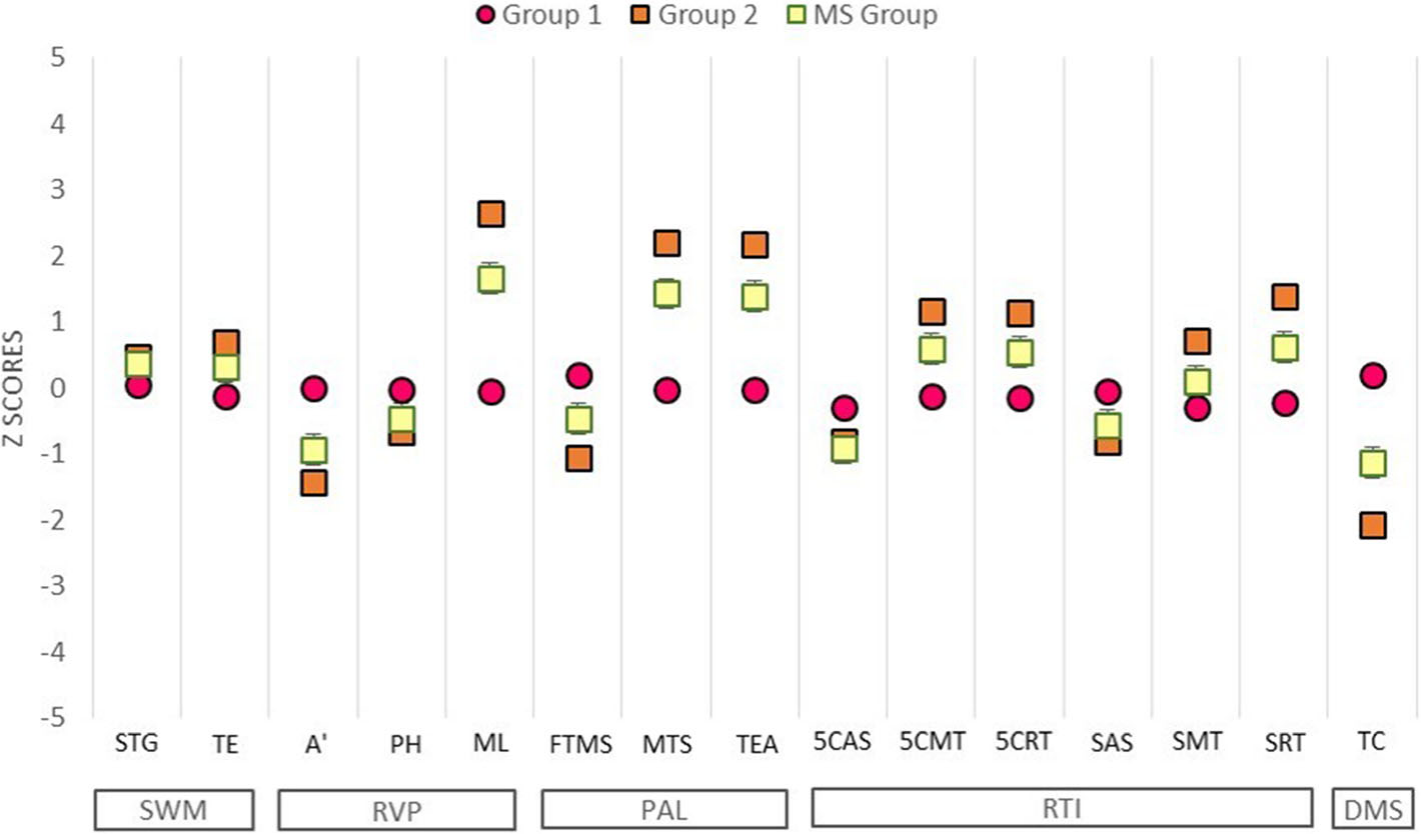
Z-scores of the mean CANTAB test performances of Group 1, Group 2, and the Multiple Sclerosis (MS) Group, with the means of the Healthy Control Group (HC) as the baseline

Although only total errors (TE; SWM), 5-choice reaction time (5CRT; RTI), simple movement time (SMT; RTI), simple reaction time (SRT; RTI), and total correct (TC; DMS) were significantly different with each other (Student’s t test; *p* < 0.05), Group 2 and MS groups had lower performance than Group 1 on the majority of CANTAB tests, as shown by the z-scores (Fig. 7). In addition, almost all MS subjects (97.1%) had, at least, z-score subtle cognitive deficits based on Healthy Control (HC) means (standard deviation ≥0.5, Table 5).

Supplementary clinical data is shown in Additional file 2: Table S2. As observed, only 4 patients showed optical neuritis one of which in Group 1 and 3 in Group 2 suggesting that optical neuritis cannot explain lower scores in CANTAB cognitive tests of MS group.

## Discussion

This study investigated the extent to which general neuropsychological pencil and paper tests and CANTAB tests, either alone or in combination, can detect subtle cognitive deficits in MS patients early in the course of their disease. Hierarchical cluster and discriminant analyses revealed that CANTAB tests could better distinguish between the cognitive performance of MS Groups than the general neuropsychological assessment. Although the present study sample is small, and the results may not be generalizable, this exploratory study strongly suggests that CANTAB test results may improve the signal-to-noise ratio and thereby distinguish the performance of subgroups of MS patients better than general paper and pencil neuropsychological tests. Thus, we suggest that the use of large-scale automated visuospatial tests to assess the information processing speed, learning, and memory on CANTAB tests may help discriminate between normal and impaired cognitive performance in MS patients.

Impairment in information processing speed (IPS) is the most common cognitive dysfunction in MS patients [7]. This impairment is associated with unemployment [24, 25], which can cause additional suffering and worse quality of life, as it affects self-esteem and overall mental and physical health and can lead to depression and somatization [26]. Neuropsychological tests, with cluster analysis, have previously been used to demonstrate in a large group of subjects that IPS and memory deficits can be used to differentiate between MS patients with versus without cognitive impairments, highlighting the central role of IPS in cognitive impairment [27]. However, the study employed individuals with a higher mean EDSS and the age range included many older patients. Furthermore, cluster analysis of event-related potentials from EEG signals and behavioral responses [28, 29] found that IPS is an early and important marker of cognitive dysfunction in MS. In this context, the Brief International Cognitive Assessment for Multiple Sclerosis (BICAMS) [30] brought together cognitive tests with distinct domains with the Symbol Digit Modalities Test (SDMT) for assessing IPS impairment, as it is sensitive to cognitive changes, correlates with brain MRI parameters, and is associated with employment status.

The Minimal Assessment of Cognitive Function in MS (MACFIMS), a 90-min overall cognitive assessment, covers more cognitive domains that are affected in MS than does the BICAMS assessment [31], but it has limited scale measurements compared to the CANTAB tests, which measure reaction times in milliseconds. However, different from BICAMS, MACFIMS or other cognitive assessments, CANTAB battery lacks validation in MS and, also, as a limitation of this study, were not compared to such validated assessments in MS to identify similar or more accurate outcomes.

Thus, in the present report, we suggest that the use of additional automated cognitive assessment tools from the CANTAB may detect subtle early cognitive dysfunction. This will help researchers develop earlier evidence-based interventions programs for cognitive rehabilitation.

To our knowledge, this is the first study to use hierarchical cluster analysis of multimodal CANTAB variables in a clinical study of cognitive dysfunction in MS patients. Consistent with previous studies, RTI measures, which reflect IPS, were the main variables in discriminant analysis, demonstrating the ability of this test to classify cognitive decline using hierarchical cluster analysis. In accordance with previous reports [32–35], learning and memory were less affected than IPS in MS patients. Thus, we suggest that PAL, DMS, and SWM test scores contribute less to cluster formation because the impact of reduced IPS is greater than the impact of impairments in learning and memory per se. Indeed, RTI and RVP contributed the most to cluster formation. We found significant differences in SWM scores in Group 1 versus Group 2, but not in the MS Group versus the HC Group. This is consistent with a previous report [35] and suggests that the CANTAB is a good choice for assessing executive function in MS.

Executive function impairment has been associated with higher EDSS score. Since the mean EDSS of the MS group utilized in this study was quite low (mean EDSS: 1.44 ± 45), we might have expected a less pronounced cognitive domain in the MS cognitive dysfunction of this sample comparing to other MS populations [36]. However, the significant impairment in RVP and RTI measures scores in low EDSS scores subjects, as presented in this study, shows not only that there is early cognitive impairment in the least disabled MS patients, but our study also reveals the power of the CANTAB assessment to detect this early impairment. In a recent report [36], CANTAB utilization without RTI or RVP tests in MS subjects displayed IPS and attention as the least prevalent cognitive domain impaired in MS, which contrasts with our findings regarding the centrality of IPS impairment in MS cognitive dysfunction, but also suggests that the utilization of IPS-sensitive CANTAB tests are, indeed, necessary.

The first studies that utilized the CANTAB in MS used the SWM and Spatial Span tests to investigate the executive function of patients with frontal lobe lesions [15]; to study deficits after acute relapse [14]; to correlate scores with magnetic resonance spectroscopy imaging [16]; and to compare cognitive dysfunction in MS subtypes [37]. Other studies investigated different aspects of MS cognition, such as memory [17] and decision making [18].

The first report of the use of the CANTAB in MS in a Brazilian population was published in 2011 [38]. That report described MS patients and patients with Duchenne muscular dystrophy as well as children and adult controls moving towards CANTAB norms in Brazil. The present study assessed cognitive dysfunction in MS patients living in the North Region of Brazil and used cluster analysis to differentiate patterns. Interestingly, patients with a benign MS subtype often perform worse on cognitive assessment tests and display a more heterogeneous pattern of cognitive dysfunction, suggesting silent deterioration of cognitive function [28, 29]. Our analysis grouped some healthy subjects with some MS patients because the MS group included both cognitively impaired and unimpaired patients, which is consistent with a previous study [28].

It is important to note that disease-modifying therapies such as interferon β-1a [39], interferon β-1b [40], and natalizumab [41] can help preserve cognitive function in MS patients. These therapies play important roles in stabilizing or delaying cognitive dysfunction in relapsing-remitting MS. Thus, patients who do not receive these therapies could experience more severe cognitive deterioration, as observed in patients with non-cognitive impaired MS patients [29]. Compared to patients taking other disease-modifying drugs or taking no drugs, patients treated with interferon-based therapy showed no statistically significant differences in cognitive performance in this study.

Finally, despite the limited associations between education and test performance in our sample, formal education was associated previously with cognitive reserve in MS patients [42], with highly educated subjects showing better performance. Thus, it is important to include multisensory and cognitive stimulation in MS clinical intervention programs.

## Conclusions

Our results suggest that the use of large-scale automated visuospatial tests, such as the CANTAB could improve the signal-to-noise ratio and reveal subtle and earlier changes in information processing speed (RTI and RVP) and learning and memory (PAL and DMS) in MS patients. This could help distinguish between normal and pathological decline in MS and contribute to the development of evidence-based individualized rehabilitation programs. Notably, most studies of CANTAB tests of MS patients have been conducted in the United Kingdom, while other countries lack normative data for CANTAB tests in MS patients. Thus, we further suggest that large-scale studies are needed in Brazil to determine whether the CANTAB can, in fact, be used as a diagnostic tool to detect cognitive impairment in MS.

## Additional files

Additional file 1: **Table S1.** Description of the cognitive tests used in this study based on the CANTAB user manual. Technical details of each cognitive test selected from the CANTAB. (DOCX 15 kb)

Additional file 2: **Table S2.** Mean values and standard errors for MS group scores on RTI, RVP, and PAL reassessment, 6–9 months later than the first evaluation. Results of selected CANTAB tests 6–9 months later than the first evaluation, to show MS patients performances evolution in the reaction time (RTI), rapid visual processing (RVP) and paired associates learning (PAL). (DOCX 12 kb)

## Abbreviations

5CAS: 5-choice accuracy score
5CRT: 5-choice reaction time
CANTAB: Cambridge neuropsychological test automated battery
DMS: Delayed matching to sample
EDSS: Expanded disability status scale
HC: Healthy control
IPS: Information processing speed
ML: Mean latency
MS: Multiple sclerosis
MSRT: Mean simple reaction time
MTS: Mean trials to success
PAL: Paired associate learning
PH: Probability of hit
RTI: Reaction time
RVP: Rapid visual processing
SAS: Simple accuracy score
SMT: Simple movement time
SRT: Simple reaction time
STG: Strategy
SWM: Spatial working memory
TC: Total correct
TEA: Total errors adjusted
WLR: Word list recognition
