# Supplementary material for: The subtleties of cognitive decline in multiple sclerosis: an exploratory study using hierarchichal cluster analysis of CANTAB results
Source: BMC Neurol. 2018 Sep 10;18:140. doi: 10.1186/s12883-018-1141-1 (PMC6131879; doi:10.1186/s12883-018-1141-1)
Supplement: Supplementary file 1 — Table S1. Description of the cognitive tests used in this study based on the CANTAB user manual. Technical details of each cognitive test selected from the CANTAB. (DOCX 15 kb) [file 12883_2018_1141_MOESM1_ESM.docx]

***Additional file 1: Table S1*. Description of the cognitive tests used in this study based on the CANTAB user manual (adapted from Bento-Torres et al.^[43]^)**

| Evaluated function | General test description | measurement (units) | assessment results |
| --- | --- | --- | --- |
| Visual sustained attention | Rapid visual information processing (RVP). A white box appears at the centre of the screen in which digits from 2–9 are presented in pseudorandom order at a rate of 100 digits/min. Subjects have to identify three-digit target sequences and respond using the press pad. | RVP latency (ms) | The mean time it takes to respond, which is a good indicator of sustained attention function. |
|  |  | RVP A' (score) | A measure of sensitivity to the target. |
|  |  | RVP probability of hit (score) | The probability of the subject responding correctly. |
| Reaction time (Processing and psychomotor speed and accuracy) | Reaction Time (RTI)  A yellow circle appears on the screen in either a single location (simple) or in one of five possible locations (five-choice). The volunteer holds down the press touch pad button until the yellow circle appears on the screen and then releases it upon detecting the stimulus presentation. Immediately afterwards, the volunteer touches the screen where the circle appeared. | RTI simple accuracy score (score) | The total number of trials in which the response is recorded as correct for assessment trials in which the stimuli appear in only one location. |
|  |  | RTI five-choice accuracy score (score) | The total number of trials in which the response is correct for assessment trials in which the stimuli appear in one of five locations. |
|  |  | RTI simple reaction time (ms) | The time it takes the subject to release the press pad button in response to stimulus onset in a single location. |
|  |  | RTI five-choice reaction time (ms) | The speed with which the subject releases the press pad button in response to a stimulus in any one of five locations. |
|  |  | RTI simple movement time (ms) | The time it takes the subject to touch the stimulus after releasing the press pad button in trials in which the stimuli appear in only one location. |
|  |  | RTI five-choice movement time (ms) | The time it takes the subject to touch the stimulus after releasing the press pad button in trials in which the stimulus is presented in one of five possible locations. |
| Learning and memory | Paired Associates Learning (PAL)  Six to eight boxes are displayed on the screen and open to reveal the contents, one at a time in a randomized order. The revealed patterns are then displayed in the middle of the screen one at a time, and the participants must touch the box in which the pattern was originally shown. Each stage may involve up to 10 trials (attempts), including the first presentation of all the shapes, followed by up to 9 repeated presentations. | PAL total errors adjusted (score) | The total number of errors across all spatial locations at all stages, with an adjustment for each stage due to a previous failure. |
|  |  | PAL mean trials to success (score) | Calculation of the trials required the subject to correctly locate all of the patterns in all stages, divided by the number of successfully completed stages. |
|  |  | PAL first trial memory score (score) | The number of patterns that are correctly located after the first trial, summed across the completed stages. |
| Spatial working memory | Spatial Working Memory (SWM)  The participant must search for a blue token in several colored boxes on the screen. The tokens are used to ﬁll an empty column on the right hand-side of the screen. The number of boxes is gradually increased from 3 to 8. The boxes’ colors and positions are changed from one trial to another to discourage the use of stereotyped search strategies. | SWM strategy (score) | An evaluation score for the strategy used to complete the task. |
|  |  | SWM total errors (score) | The number of times the volunteer selects a box that does not contain a blue token. |
