# Supplementary material for: The subtleties of cognitive decline in multiple sclerosis: an exploratory study using hierarchichal cluster analysis of CANTAB results
Source: BMC Neurol. 2018 Sep 10;18:140. doi: 10.1186/s12883-018-1141-1 (PMC6131879; doi:10.1186/s12883-018-1141-1)
Supplement: Supplementary file 2 — Table S2. Mean values and standard errors for MS group scores on RTI, RVP, and PAL reassessment, 6–9 months later than the first evaluation. Results of selected CANTAB tests 6–9 months later than the first evaluation, to show MS patients performances evolution in the reaction time (RTI), rapid visual processing (RVP) and paired associates learning (PAL). (DOCX 12 kb) [file 12883_2018_1141_MOESM2_ESM.docx]

| Cognitive Reassessment N^o^ of patients (n; %) | Mean Time for Reassessment (months ±SD) | Major overall score outcome decline* (n; %) | Major RTI score outcome decline* (n; %) | Major RVP score outcome decline* (n; %) | Major PAL score outcome decline* (n; %) | Major RTI+RVP scores outcome decline* (n; %) | Patients displaced in cluster analysis after reassessment | Optical neuritis in Group 1 (n; %) | Optical neuritis in Group 2 (n; %) |
| --- | --- | --- | --- | --- | --- | --- | --- | --- | --- |
| 27 (77.14 %) | 9.39 ± 3.59 | 13 (48.14%) | 8 (29.62%) | 1 (3.7%) | 7 (25.92% | 8 (29.62%) | None | 1 (2.27%) | 3 (13.04%) |

***Additional file 2: Table S2. Mean values and standard errors for MS group scores on RTI, RVP, and PAL reassessment, 6 – 9 months later than the first evaluation.***
